# Efficient Communication Scheme for Bluetooth Low Energy in Large Scale Applications

**DOI:** 10.3390/s20216371

**Published:** 2020-11-08

**Authors:** Maciej Nikodem, Mariusz Slabicki, Marek Bawiec

**Affiliations:** 1 Faculty of Electronics, Wrocław University of Science and Technology, Wybrzeże Wyspiańskiego 27, 50-370 Wrocław, Poland; marek.bawiec@pwr.edu.pl; 2 Institute of Theoretical and Applied Informatics, Polish Academy of Sciences, ul. Bałtycka 5, 44-100 Gliwice, Poland; mslabicki@iitis.pl

**Keywords:** BLE, advertisement, opportunistic sensing, real-life evaluation, simulations, active scanning, intermittent operation, energy efficiency

## Abstract

The use of Bluetooth Low Energy (BLE) in the Internet-of-Things (IoT) applications has become widespread and popular. This has resulted in the increased number of deployed BLE devices. To ensure energy efficiency, applications use connectionless communication where nodes broadcast information using advertisement messages. As the BLE devices compete for access to spectrum, collisions are inevitable and methods that improve device coexistence are required. This paper proposes a connectionless communication scheme for BLE that improves communication efficiency in IoT applications where a large number of BLE nodes operate in the same area and communicate simultaneously to a central server. The proposed scheme is based on an active scanning mode and is compared with a typical application where passive scanning mode is used. The evaluation is based on numerical simulations and real-life evaluation of a network containing 150 devices. The presented scheme significantly reduces the number of messages transmitted by each node and decreases packet loss ratio. It also improves the energy efficiency and preserves the battery of BLE nodes as they transmit fewer radio messages and effectively spent less time actively communicating. The proposed connectionless BLE communication scheme can be applied to a large variety of IoT applications improving their performance and coexistence with other devices operating in the 2.4 GHz band. Additionally, the implementation complexity and costs of the proposed communication scheme are negligible.

## 1. Introduction

Bluetooth Low Energy (BLE) is a popular low-power radio communication technology available in contemporary smartphones, tablets and computers. Developed in the early 90 s, it has undergone significant changes and improvements. Among others, the current 5.2 version [1,2,3] allows for medium range communication, different throughput, angle of arrival/departure estimation, and ultra low-power operation especially in connectionless mode. These improvements expanded the range of possible applications and motivated the use of BLE in the Internet-of-Things (IoT) systems, especially in monitoring and opportunistic sensing applications [4,5]. In such applications, the BLE nodes take periodic measurements and transmit them to the Internet-enabled gateway (also called scanner). The nodes can transmit data using either connected or connectionless (also called advertisement) communication modes. The latter is preferred when the number of nodes is large, low-power operation is crucial, and the amount of data to be transmitted is limited [5]. However, connectionless mode enables only node-to-gateway data transmission and does not provide reliability mechanisms that are available in connected mode. To overcome this drawback, the nodes transmit data periodically assuming that, despite the possible interference, the scanner will eventually receive the data successfully. Periodic transmissions ensure transient interference do not obstruct data transmission. However, they increase the number of messages transmitted in the radio channel, collisions, and energy consumption. To address a wide range of applications, connectionless communication has two variants: passive and active scanning. One of the most important differences between them is the number of radio messages exchanged between the node and the gateway; one and three messages for passive and active scanning, respectively.

So far, many authors (e.g., [6,7,8,9,10]) focused on passive scanning and determining optimal settings for the nodes and scanners (namely advertisement interval, scan window, and scan interval) when a large number of nodes is deployed in small area. They have analysed the impact on the energy consumption, effective data throughput, and detection delay (the time until the scanner correctly receives the first data packet from a node), and proposed methods to choose settings for different scenarios. All these approaches attempted to improve communication through determining the best settings for the nodes in the network while simultaneously keeping the detection delay as low as possible, for a given number of BLE devices. Unfortunately, these approaches do not take into account that the bandwidth available in the 2.4 GHz frequency is shared among different BLE networks and other radio technologies (e.g., WiFi, IEEE 802.15.4). Therefore, in real-life scenarios, not only the expected performance of the node may deteriorate (e.g., [11,12,13,14]), but also the operation of other radio technologies may be significantly affected. Moreover, setting fixed BLE communication parameters is not suited for applications where the number of nodes/scanners changes over time and the parameters should be adjusted accordingly. Achieving this, however, requires the nodes to track the number of active devices or detect interference in the radio channel. This is challenging, imposes additional energy costs, and may affect the lifetime of the nodes.

Active scanning has gained less attention in the literature (e.g., [15,16]) because the increased number of messages transmitted adversely affects communication performance and intensify aforementioned issues. However, as we present in this article, the three message communication scheme can be used by the scanner to acknowledge correct reception of the data sent by the node. When this acknowledgement is received, the node is informed that it may stop further periodic transmissions, reducing the congestion in the communication channel. As presented, the proposed communication scheme allows to reduce overall number of radio messages, improve the efficiency of data communication in dense networks, and reduce nodes’ energy consumption. Moreover, the efficiency of acknowledgement transmission depends both on the number of BLE devices and other 2.4 GHz radios. Therefore, the three message scheme automatically adjusts the node’s operation to variable channel conditions. In any case, the node will suspend the transmission once the acknowledgement is received. The ability to suspend or reduce frequency of transmission also improves the coexistence with other radios, as they will experience less interference and will be able to use more of the available bandwidth.

The proposed communication scheme aligns well with various approaches that aim to reduce the amount of data to be transmitted. For example, adaptive sampling allows to dynamically adapt sampling rate to the variability of the signal, and trade-off energy savings with data precision [17]. The volume of data transmitted across the network can also be reduced based on the run-time knowledge of the measurement stream evolution and its variability [18]. This information allows to build a model of the measurement stream and transmit only when the model needs to be updated. Based on the model the receiver can calculate the next measurements without the need for additional transmission.

This paper presents and evaluates the aforementioned BLE communication scheme using real-life experiments and numerical simulations. We analyse the impact of connectionless communication, active scanning and intermittent transmission on the performance of BLE communication. The contribution of this paper includes:

- experimental evaluation of a large number of BLE devices operating in real-life environment in the presence of mutual and external interference, using connectionless communication for data transmission,
- results of numerical simulations to assess the impact of active scanning and suspending advertisements (upon reception of a scan request) on communication and energy efficiency,
- demonstration of the applicability of the connection-less active scanning BLE mode to large-scale opportunistic applications.

The paper is organised as follows. Section 2 presents previous results and analyses the BLE communication performance and energy efficiency for deployments where a large number of BLE nodes coexist in a small area. Section 3 shows more details on the passive and active operation of BLE scanners and details of the proposed communication scheme. Section 4 describes real-life experimental setup, evaluation scenarios and defines parameters of interest that are used to assess the benefits of the proposed communication scheme. Section 5 summarizes the results of the experiments and simulations.

## 2. Related Work

Because the number of BLE devices using connection-based communication is limited [19,20], the practical IoT applications have to use connection-less mode. In this mode, the nodes (peripherals) and scanners (central devices) use three types of messages: advertisement (ADV), scan request (SCAN_REQ) and scan response (SCAN_RESP). Advertisement messages are sent periodically by a BLE node that wants its presence to be detected by scanners. When a scanner operates in active scanning mode and receives the advertisement, it can send the scan request which triggers the node to transmit the scan response. Scanners operating in passive mode do not send scan requests.

A number of papers focus on collisions in the communication channel, energy efficiency and node discovery delay, i.e., time from the first advertisement transmitted to the first time the scanner correctly receives one advertisement (but not necessarily the first one) and notices the presence of a node. This includes analyses of different versions of the BLE standard [15,21], operation patterns including connection-less and connection-based modes [5,6,22,23,24,25], passive and active scanning [15,16], and various choice of communication parameters (e.g., advertisement/scan/connection interval, scan window) [6,7,8,15,21,23].

Appropriate selection of BLE communication parameters allows to trade-off power consumption with device discovery delay and data throughput. Works by Shan et al. [7,8] presented a method to determine the best value of the advertisement interval to minimize the discovery delay of all surrounding BLE advertisers, by a single scanner, operating in passive mode. They also analyzed the effect of the advertisement interval on the energy consumption. The analytical model and simulation results show that the appropriate choice of advertisement interval, for a given number of BLE advertisers, can minimize the discovery time and energy consumption significantly. Therefore, the value of the interval ought to be adjusted to the number of nodes in the network; in general, it should increase with the number of nodes. The results also present that in continuous scanning mode the impact of scanner parameters (scan window and scan interval) on advertisement reception is negligible if both exceed 0.1 s, which is the case for most applications.

Renzel et al. [23] focused on human interaction with smart objects such as smart locks. They proposed an adaptive BLE discovery procedure in which frequency of advertisement transmission is adjusted to the past user behaviour in order to find trade-off between responsiveness (detection delay) and system energy consumption. The results show that adapting advertisement interval may significantly reduce the energy consumption and average discovery time, compared to the use of fixed setting. The article considers only sparse deployments and does not take into account collisions in the communication channel. However, it shows that appropriate use of mechanisms supported in BLE specification can significantly improve device operation, including discovery delay and energy efficiency.

Further energy savings can be achieved if BLE radios are used only when needed and turned off otherwise. Venazi et al. [24] focused on an application where the nodes can decide their location and adjust operation accordingly. They proposed to use dual interface radios (WiFi and BLE) and a fog architecture for smart management of BLE scanners and advertisers. Fog devices communicate with the nodes using lightweight protocols over WiFi, detect presence of the nodes, and manage BLE scanning/advertising accordingly. The fog architecture eliminates unnecessary periodic broadcasting of advertisements and scanning when the distance between the devices exceeds the BLE communication range. However, the use of additional communication technology (WiFi) as well as the need to decide the node location (proximity) is serious disadvantage, that affects system complexity and power efficiency of the nodes. As a result, careful consideration is required before it is applied in real systems.

The article by Ghamari et al. [25] examines collisions and energy costs when a large number of nodes transmit advertisement packets simultaneously. The paper develops an advertisement collision model that allows to estimate the collision probability as a function of communication parameters (advertisement interval) and the number of communicating nodes. The results show that reducing the advertisement intervals significantly increases packet collisions probability. This affects the amount of the advertisements received, and increases node’s energy consumption. Unfortunately, the simplified model of the collisions and small-scale real-life validation (7 devices) is a limitation of this work.

Connectionless BLE communication is often used in opportunistic sensing applications [4,5,26,27]. In those applications, sensing devices broadcast the information inside the payload of advertisement messages. Advertisements are coincidentally received by scanners which are gateways with Internet connectivity, and can forward the information to servers. This approach enables various monitoring applications (e.g., [26,27]) and eliminates the need for a dedicated Internet connectivity in each device. Additionally, it can benefit from the fact that most of the contemporary devices (including smartphones and single board computers) have build-in BLE and Internet connectivity, and can easily act as gateways. This enables those devices to establish a crowdsensing application [4] and act as gateways for forwarding the data to the Internet. Aguilar et al. [5] investigated the feasibility and the trade-offs of using advertisement-based and connection-based opportunistic sensing. They have presented, that when the amount of data to transmit is small the connectionless approach enables improvement in energy consumption and improves the lifetime of the sensor device.

BLE nodes can simultaneously work in connection-based and connectionless mode. Therefore, some papers address such scenarios, e.g., the article by Del Campo et al. [6] analyses the system dedicated for assisted ageing living (AAL) scenario where some of the sensors are continuously connected and some are sending their measurements only form time to time, using advertisement messages. They have proposed a tool to support selection of BLE device parameters in the system to ensure practical discovery latency and avoid overlaps between scanning and connection phases. Unfortunately, the presented analyses and results are for a single scanner only and do not consider active scanning.

Perez-Diaz De Cerio et al. [22] have analysed discovery performance using real BLE devices, when BLE scanners operate in passive mode. Authors distinguished two types of scanner operation, identified and characterized gaps in the scanning procedure that result from the non-idealities of real devices. The gaps shorten effective scanning time and affect performance of node discovery. The article evaluates detection procedure through numerical simulations and experimental measurements using limited number of advertising nodes. The results show that the simulation closely matches the measurements. This work was extended by Hernandez-Solana et al. [15] who analysed the performance of BLE device discovery when scanners operate in active mode. Extending the results presented in [22] they have characterized operation of real BLE scanners, and derived state diagrams for them. Those diagrams provide detailed insight into operation of BLE scanners in active scan and the backoff procedure. The article also derives mathematical models and software simulator to estimate collision probabilities and expected discovery time for dense deployments. The results show that the use of active scanning and interrupted advertisement transmission can reduce the discovery time, and energy consumption.

Another paper by the same authors [16] addresses the performance of backoff procedure implemented in active scanning. The procedure is intended to minimize collisions of scan request messages transmitted by several scanners in response to a single advertisement received. The scanner adjust the backoff value based on the scan response reception efficiency. This procedure is evaluated for each scan request transmitted by the scanner. The article also presents a modification of the backoff algorithm that improves scanner coexistence by differentiate situations when scan response is not sent (due to loss of scan request) and when scan response is sent but cannot be correctly received due to collisions. The resulting improvement allows to keep backoff intervals lower and further shorten node detection latency.

## 3. Communication Scheme

The communication scheme analysed in this paper benefits from connectionless BLE communication and active scanning. For simplicity we limit analysis to legacy advertising (as defined in BLE standard [1]) however presented results can be extrapolated to other types of protocol data units (PDUs), including extended advertisements.

### 3.1. Passive and Active Scanning

In connectionless communication mode the BLE nodes (advertisers) periodically broadcast advertisement (ADV) packets that can be coincidentally received by scanners. Depending on nodes and scanners configuration the devices may exchange either one or three radio messages (Table 1).

Single message mode (ADV only) is used when scanner operates in passive scanning mode or the node does not support scanning operation. Single message allows for unconfirmed, one way communication from the node to the scanners (several BLE scanners may receive the same ADV if they are within the communication range). Because the ADV message may contain up to 31 bytes of data, they can be used for application specific data transmission e.g., in opportunistic sensing applications [4,5].

**Figure 1:**
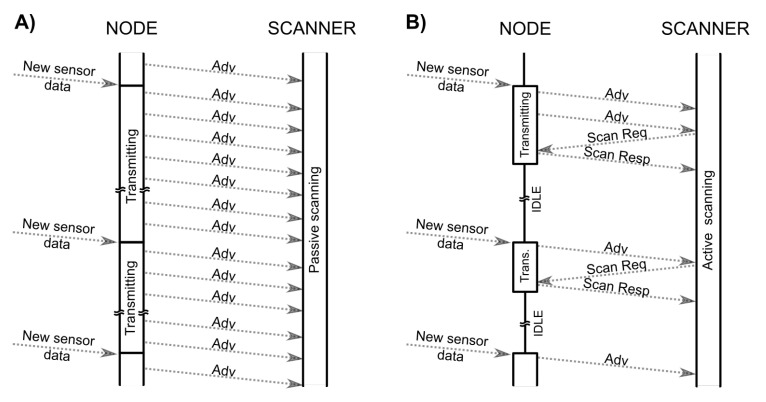
BLE connectionless communication using (**A**) passive and (**B**) the proposed communication scheme. In the proposed scheme the reception of SCAN_REQ allows the node to terminate ADV transmission in order to preserve energy and improve spectrum sharing.

Three message mode requires the scanner to operate in active scanning mode an the node to support scanning procedure. In three message mode the scanner may transmit the SCAN_REQ message after receiving the ADV. Frequency of SCAN_REQ depends on the content of the ADV received as well as a backoff procedure implemented in the scanner [1,15]. In general the active scanner shall sent SCAN_REQ when content of the ADV changes or after reception of at most 256 ADV messages (256 is the highest value of the backoff counter). The SCAN_REQ message does not contain any data and, after it is received by the the node, it initiates transmission of a SCAN_RESP message. The structure of SCAN_RESP is similar to the structure of ADV and it may contain another 31 bytes of data. In this operation mode the radio packets are transmitted back and forth, but the data is still transmitted from the node to the scanner. Moreover, while ADV messages can be received by all the scanners, the SCAN_RESP are received only by the scanner that has sent corresponding SCAN_REQ message. Although, the SCAN_REQ message is not transmitted for every received ADV and it cannot contain application specific data, it can be used as an acknowledgement to confirm correct reception of the ADV by the scanner. This allows to design a communication scheme that uses three message communication in order to terminate ADV transmission as soon as the transmitted data is received. This improves communication efficiency, decrease the interference in the communication channel, and reduce node’s energy consumption. Figure 1 presents the difference between the connectionless communication using passive and the proposed communication scheme.

Table 2 presents the symbols used in the remaining part of the article and used to describe the operation of BLE communication and the proposed scheme.

### 3.2. The Proposed Communication Scheme

**Figure 2:**
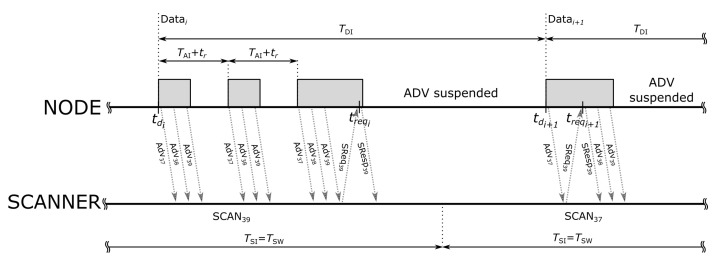
The three message communication scheme. The gray color marks the advertisement events that contain subsequent transmission of three ADVs on channels 37, 38 and 39. The node responds with SCAN_RESP and suspends further advertisement events when the first SCAN_REQ is received.

In the proposed three message communication scheme (Figure 2) the SCAN_REQ provides feedback to the node on the correct reception of the ADV and the data contained in the payload. Therefore, when the node has data to be transmitted it may split it into chunks of at most 31 bytes. Each chunk is then broadcasted periodically inside the payload of ADV messages. After reception of these messages the scanner will eventually send a SCAN_REQ message. When SCAN_REQ is received by the node, it is interpreted as a confirmation that at least one scanner (the one that sent the SCAN_REQ) has correctly received the data. The node may then disable further ADV transmission until new chunk of data is available. We call this operation an intermittent mode as nodes stop and restart ADV transmission when SCAN_REQ is received and new data is available.

Because the scanners run a backoff procedure before sending the SCAN_REQ message (cf. [1,16]), therefore, not every received ADV initiates the exchange of SCAN_REQ and SCAN_RESP messages. The transmission of SCAN_REQ can also be disturbed by the interference in the radio channel. Consequently, the node may need to transmit several ADV messages before correct reception of the data is confirmed with SCAN_REQ.

According to the BLE specification [1], the node that receives SCAN_REQ has to respond with SCAN_RESP message that may also contain application-specific data. However, when operating in the intermittent mode the node sends SCAN_RESP only once and terminates ADV transmission making it impossible for the scanners to sent another SCAN_REQ. Therefore, the data contained in the SCAN_RESP is transmitted only once, without confirmation. The transmission of such data is prone to interference, communication errors, and loss.

## 4. Evaluation Scenarios

Efficiency of the proposed communication scheme was evaluated in a simulations and an experimental setup imitating a large scale opportunistic sensing application; a number of end-devices periodically transmit measurement data to a central unit using connectionless BLE communication. In such applications (e.g., [26,27]) a large number of BLE devices are deployed in a small area resulting in increased radio communication interference and adversely affecting the efficiency of data transmission.

### 4.1. Experimental Setup

To model dense deployment we used 150 BLE devices broadcasting advertisements every 250 ms. The advertisement’s payload was 31 bytes including a sequence number that has changed every data interval τ which was set to 10 s. The data interval was used to imitate measurements taken by real nodes and enable analysis of data transmission efficiency for different values of τ. The nodes were broadcasting advertisements with PDU type set to ADV_IND enabling active scanners to send SCAN_REQ messages. The nodes were responding with fixed SCAN_RESP message of 10 bytes when SCAN_REQ was received. The nodes used in a test were based on nRF 52832 System-on-Chip (Nordic Semiconductor, Trondheim, Norway) which were running a simple communication application developed using SDK v.14.2 from Nordic Semiconductor They were deployed in small laboratory (approx. 20 m${}^{2}$) and sent messages with a transmission power of 0 dBm.

The data were received by a scanner running in continuous scanning mode (i.e., scan window and scan interval parameters were set equal) in either active or passive mode, depending on the scenario. Because our goal was to imitate real-life sensing application we decided to use RaspberryPi (RPi) as scanners. These are low-cost and very popular devices with WiFi connectivity, integrated BLE radios (RPi Zero W, and RPi 4) or supporting BLE dongles (RPi 3 and earlier). Because RPis are quite limited in computational and communication performance, the results of our experiments can be considered as a lower bound of what can be expected if more efficient scanners are used. The scanners were storing measurements in random-access memory to minimize the impact of the operating system and SD card underperformance on the results of the experiments.

As part of the preparation for the actual experiments we have verified how different RPis perform as sniffers. These tests targeted assessment of different hardware setups (e.g., with/without BLE dongles) and configurations (e.g., hardware/software whitelisting of devices) on the communication performance. Based on the results we have used two sniffers (S1 and S2) running on RPi Zero W devices and operating in different modes depending on the test scenario (Table 3). Additionally, we have used a PC computer as the third sniffer (S3) to monitor the execution of the experiments. This sniffer was always running in passive mode. In order to understand operation of the nodes, we have connected one advertiser to the PC computer and logged its activities including timestamps, changes of the transmitted data (e.g., sequence number) and reception of the SCAN_REQ messages. Due to the limitations of the node’s SDK, that does not provide callbacks for ADV and SCAN_RESP transmission, these events could only be inferred from the available logs and configuration parameters (e.g., advertisement interval).

### 4.2. Parameters

The efficiency of the proposed scheme is assessed with Packet Loss Ratio (PLR) and Data Reception Ratio (DRR). The PLR is a measure that takes into account both the collisions in the communication channel as well as the underperformance of real-life scanners that may not be able to correctly receive all the packets, even if there is no collision in the radio channel. The PLR is calculated as a ratio of the number of ADV packets received by the scanner to the number transmitted by the advertising node. Because the scanner concurrently listens on a single advertisement channel, the PLR takes into account only ADV messages that are transmitted on the same channel. Therefore, the number of packets transmitted by a node ($N_{\mathrm{TxPacket}}$) equals the number of advertisement events, which can be estimated from the value of advertisement interval ($T_{\mathrm{AI}}$) and the time node was transmitting the ADV messages ($T_{\mathrm{active}}$):

(1) $$N_{\mathrm{TxPacket}}=\frac{T_{\mathrm{active}}}{T_{\mathrm{AI}}}.$$

For nodes operating in continuous mode the $T_{\mathrm{active}}$ is constant and equal to experiment duration ($T_{E}$). On the other hand, for nodes operating in intermittent mode it is a sum of active times in each data interval i.e., times from the beginning of *i*th data interval ($t_{d_{i}}$) to the reception of the first SCAN_REQ in this data interval ($t_{{\mathrm{req}}_{i}}$):

(2) $$T_{\mathrm{active}}=\left\{\begin{array}{cc} T_{E} & \mathrm{for}\;\mathrm{nodes}\;\mathrm{in}\;\mathrm{continuous}\;\mathrm{mode},\\ \sum_{i}\left(t_{{\mathrm{req}}_{i}}-t_{d_{i}}\right) & \mathrm{otherwise}, \end{array}\right.$$

where *i* iterates over all data intervals. Finally, the PLR for a node is estimated as:

(3) $$\mathrm{PLR}=1-\frac{N_{\mathrm{RxPacket}}}{N_{\mathrm{TxPacket}}},$$

where $N_{\mathrm{RxPacket}}$ denotes the number of advertisements received by the scanner.

In contrast to PLR, the DRR measures the efficiency of data transmission (i.e., sequence numbers in the experiments) and is defined as the ratio of the number of received ($N_{\mathrm{RxData}}$) and transmitted ($N_{\mathrm{TxData}}$) data:

(4) $$\mathrm{DRR}=\frac{N_{\mathrm{RxData}}}{N_{\mathrm{TxData}}}.$$

Because the transmitted data can change at different intervals, we introduce τ as a data interval and estimate the individual Data Reception Ratio (DRR${}_{\tau }$) for each τ. This parameter is similar to *x* second success ratio defined by Harris et al. [26], and is defined as the ratio of the number of received data versus transmitted data when the data (i.e., sequence numbers in the experiments) changes every τ seconds. The experimental evaluation of DRR${}_{\tau }$ for all values of τ is not feasible, therefore it is estimated from the experiments where τ=10 s. For τ<10 s the DRR${}_{\tau }$ is a ratio with the denominator that equals the number of nodes in the experiment (*N*), and the nominator depending on the operation mode of the advertisers. For nodes in the continuous mode, the nominator equals the number of nodes from which ADV were successfully received by the scanner within data interval ($N_{Rx,\tau }$). For intermittent mode it is the number of nodes for which the time from the beginning of the data interval to the reception of first SCAN_REQ (i.e., $t_{\mathrm{req}}-t_{d}$) is smaller than τ:

(5) $${\mathrm{DRR}}_{\tau }=\left\{\begin{array}{cc} \frac{N_{Rx,\tau }}{N} & \mathrm{for}\;\mathrm{nodes}\;\mathrm{in}\;\mathrm{continuous}\;\mathrm{mode},\\ \; & \\ \frac{\#\left(t_{\mathrm{req}}-t_{d}<\tau \right)}{N} & \mathrm{otherwise}. \end{array}\right.$$

Estimation of DRR${}_{\tau }$ in intermittent mode requires to monitor the operation of the node. Because monitoring large number of nodes was infeasible, therefore, it was done for a single, randomly selected node. The results of real-life experiments were verified with computer simulations where it was possible to monitor operation of every node and conduct more detailed analysis of DRR${}_{\tau }$ in intermittent mode.

### 4.3. Experimental Scenarios

Table 3 lists sniffer settings for the scenarios verified experimentally. The first scenario is used to assess performance of typical opportunistic sensing BLE applications when the nodes continuously transmit advertisements and scanners passively listen for those messages. In the second scenario the S1 sniffer is switched to active mode but the devices still broadcast the advertisements continuously. The devices are receiving the SCAN_REQ messages from S1 but do not suspend periodic transmission of ADV. The third scenario is similar to the previous one—the devices continuously broadcast advertisements but two sniffers (S1, S2) are scanning actively and can transmit SCAN_REQ messages. The fourth scenario is also similar to the second but when SCAN_REQ is received the nodes suspend ADV transmission until new data (new sequence number) is to be transmitted (i.e., the devices restart periodic ADV transmission every τ = 10 s).

The motivation for the choice of evaluation scenarios is to compare the impact of active scanning and suspension of ADV transmission on the performance of opportunistic sensing applications. Scenarios 1 and 2 enable to assess the impact of active scanning (i.e., increased number of radio transmission due to additional SCAN_REQ and SCAN_RESP messages) through the comparison of advertisement reception by scanners. Scenarios 2 and 3 show the impact of the number of scanners operating in active scanning mode, while scenario 4 shows the effect of intermittent advertisement transmission.

### 4.4. Simulations

It is infeasible to estimate all parameters of interest in real-life experiments for different number of nodes operating in intermittent mode, and various settings of BLE parameters (e.g., advertisement and data intervals). Therefore, we have developed a simulator (https://mariuszslabicki.github.io/pytooth/) to model behaviour of the BLE devices and analyse how intermittent mode affects communication efficiency compared to continuous transmission of advertisements. The model of the node and its operation is derived from the BLE specification [1] and power profiles presented by the manufacturer of the nodes (namely nRF 52832 by Nordic Semiconductor). The model assumes that ADV messages are transmitted on all three advertising channels (37, 38 and 39) and takes into account delays and randomness that are typical to real nodes. The model of the scanner is based on the results presented in [15,22]. We designed the model based on the state diagrams for the Type 2 scanners [22] and extended it with a back-off procedure for transmission of SCAN_REQ messages, as defined in BLE v5.2 standard [1].

We have used the simulator to analyse influence of intermittent mode on PLR and DRR, and draw more general conclusions regarding the benefits of the intermittent operation. Simulation results were also compared with the results of real-life experiment scenarios, presented in Table 3. The evaluation (Table 4) was done for a single scanner (operating in either passive or active mode), various number of nodes in the network, and different values of parameters (continuous and intermittent operation mode, advertisement, and data intervals). In each simulation all the nodes in the network had the same settings (e.g., intermittent mode, advertisement interval of 250 ms, and data interval of 10 s) but their operation were not synchronised and independent from each other. To reproduce real-life scenarios, the nodes were started at different time instants randomly selected from the range 0 to the length of the data interval.

Simulation scenario 1 (Table 4) was used to estimate the impact on PLR for networks of different sizes (ranging from ten to 1000 nodes), nodes broadcasting advertisements at different intervals (i.e., advertisement interval varied from 100 ms to 1 s), and fixed data interval of 10 s. Second simulation scenario was run to compare the DRR${}_{\tau }$ for different data intervals (ranging from 1 to 10 s), continuous and intermittent operation modes, different number of nodes in the network, and fixed advertisement interval of 250 ms.

## 5. Results

The proposed intermittent communication scheme was evaluated in real-life experiments for the network of 150 nodes and selected values of their parameters. Computer simulations where verified against these results and then used to draw more general conclusions regarding the possible improvement in using active scanning and intermittent operation of BLE nodes.

### 5.1. Results of Real-Life Experiments

Table 5 presents results for a single node in different scenarios. In scenarios 1–3 the node operated in continuous mode sending 40 ADV messages in each data interval of length τ = 10 s. The scanners were able to receive from 1 to 32 messages in different data intervals depending on the scenario. This means that in each scenario and for every scanner (S1, S2 and S3) the effective DRR equals 1—i.e., all sequence number sent from this node were correctly received. The S3 scanner outperformed S2 and S1 which results in higher PLR values and higher numbers of received ADV packets ($N_{\mathrm{RxPacket}}$). Different values for each scanner are due to the fact that S3 was running on a PC computer while S1 and S2 were RasperryPi–based. The presence of scanners operating in active mode (scenario 2 and 3) increases PLR values compared to only passive scanning (scenario 1) which is a consequence of additional SCAN_REQ / SCAN_RESP messages that are exchanged between the nodes and active scanners.

Results differ significantly in the scenario 4 where all the nodes terminated ADV transmission upon reception of SCAN_REQ from the scanner S1. Intermittent operation significantly lowers the number of ADV packets transmitted by each node in data interval—as presented in Table 5, the median of $N_{\mathrm{TxPacket}}$ dropped from 40 to 1. On the one hand this drop reduces the likelihood of correct ADV reception by passive scanners and causes the DRR for S2 and S3 to drop. On the other hand, the DRR for S1 was still 1, and the efficiency of transmission for all the nodes improved (i.e., the values of PLR dropped). This is due to lower collisions in the communication channel and lower load of the scanners.

**Figure 3:**
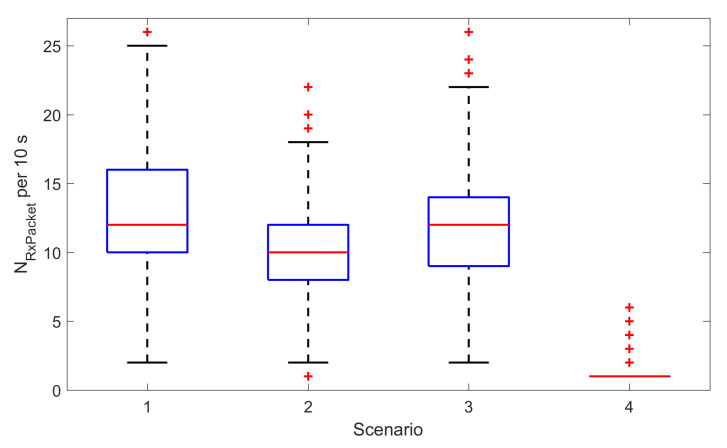
The number of ADV packets received ($N_{\mathrm{RxPacket}}$) by the S1 scanner from a single node in all data intervals, for different evaluation scenario. The number of packets received in scenario 4 drops significantly due to active scanning and intermittent operation of the node.

Comparing performance of S1 in all the scenarios (Figure 3) it can be seen that active scanning with intermittent transmission of advertisements allows to reduce the number of messages in the radio channel. Consequently, this transmission mode improves the reception of packets (cf. PLR in Table 5), and maintain the same DRR for the active scanner.

**Figure 4:**
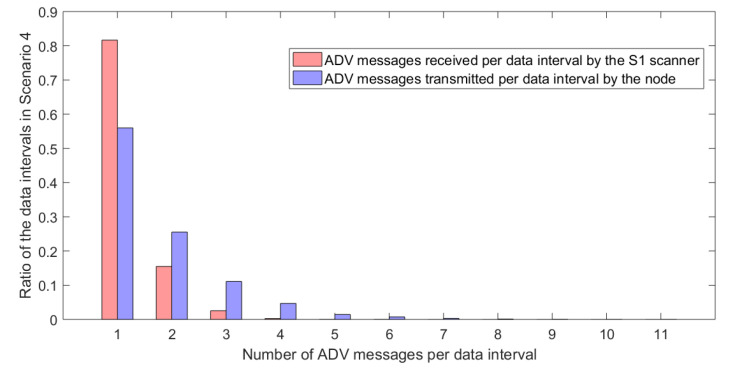
The number of ADV packets transmitted by a single node (blue) and received by the S1 scanner (red) for all data intervals in the experiment scenario 4.

Figure 4 presents the plot of the number of ADV packets transmitted by a single node and received by S1 scanner for all data intervals in the scenario 4. For over 55% of the data intervals the node has sent only one ADV message and turned off the advertisements—this means both the ADV and SCAN_REQ messages were sent successfully in the first attempt for 55% of the data intervals. On the other hand, for over 80% of data intervals the scanner S1 has received a single ADV message. Higher ratio for the scanner is related to the fact that the scanner has lost some of the ADV before the one that was correctly received. It also shows that once the ADV was received the SCAN_REQ was transmitted back and its transmission was effective—otherwise the node would have kept sending another ADV messages and cause the scanner to receive more then one ADV message.

Table 6 presents statistics of DRR and the number of received messages for all scanners in different scenarios. When all nodes were operating in continuous mode (scenarios 1, 2 and 3) the DRR was close to 1 in all cases, except for S1 and S2 scanners that have missed a few data for some of the nodes causing the minimum value of DRR to drop slightly below 1. In scenario 4 the active scanner (S1) received all the data from all nodes (DRR = 1). The DRR is lower for the passive scanners (S2 and S3) because nodes in the intermittent mode transmit small number of ADV per each data interval (cf. Table 5). In fact, in this scenario the amount of ADV and SCAN_RESP messages received by all the scanners are the order of magnitude lower compared to scenarios 1–3.

In all scenarios, the number of SCAN_RESP messages received by the active scanner, is almost half of the number of advertisements received. This value shows that the scanners send the SCAN_REQ messages frequently. Assuming the scanners implement the backoff procedure this means that small values of backoff count are maintained during the whole test. This suggests that the success ratio of SCAN_RESP reception is high (otherwise the backoff counter would increase and consequently the number of transmitted SCAN_REQs and received SCAN_RESPs, would drop).

**Figure 5:**
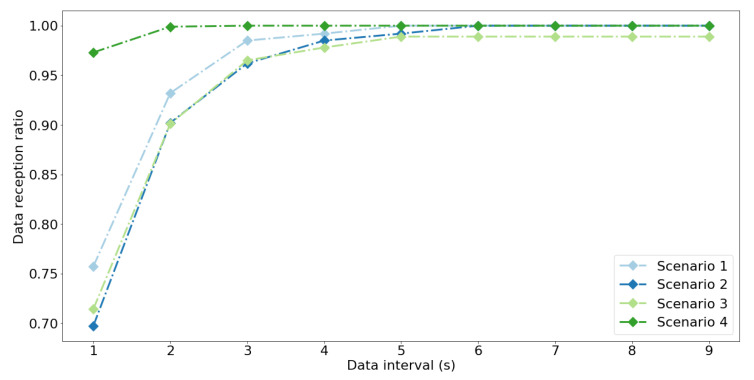
Median of DRR${}_{\tau }$ estimated from real-life experiments for the scanner S1, various scenarios and data intervals τ < 10 s.

Based on the results of the experiments we have estimated the expected values of DRR${}_{\tau }$ for different values of data interval τ (Table 7, Figure 5). The minimal value of DRR${}_{\tau }$ exceeds 0.99 only in scenarios 1 and 4. However, in scenario 1 this happens for data intervals greater then 8 s, while for scenario 4 this threshold equals 2 s. This confirms that using active scanning and continuous operation for nodes (scenario 2 and 3) has an adverse effect on the communication performance. However, allowing the nodes to suspend the transmission of ADV messages after SCAN_REQ is received (scenario 4) allows to significantly improve effectiveness of data transmission.

### 5.2. Simulation Results

Simulation analyses were done for two sets of scenarios (Table 4): passive scanner with continuous transmission of ADV messages by nodes (continuous operation), and active scanner with intermittent transmission of ADV messages by nodes (intermittent operation). The simulations of active scanner with nodes continuously transmitting ADVs is not presented because the results are worse than for continuous operation; in active scanning, the scanner transmits SCAN_REQ messages, and nodes respond with SCAN_RESP that increase the flood of ADV messages generated by the continuously transmitting nodes. This leads to the increased number of messages in the radio channel, and lowers the performance of the communication below values achieved for continuous operation with passive scanner. Similarly, simulations of passive scanner and nodes terminating ADV transmission is also omitted because the results are equivalent to continuous operation; in passive scanning, the scanner does not send SCAN_REQ messages and the nodes cannot suspend ADV transmission. For clarity, and because the variance is small the presentation of the results includes only the median values.

**Figure 6:**
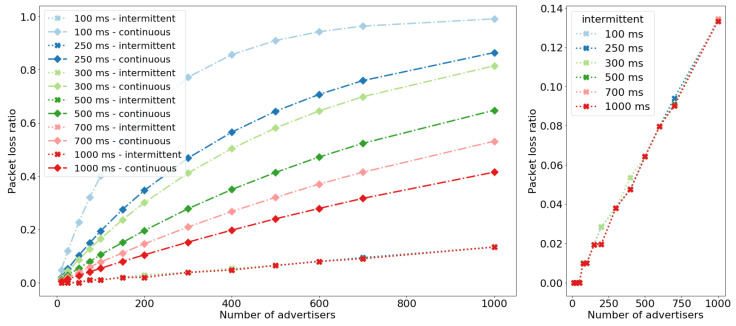
Median of the Packet Loss Ratio for continuous and intermittent operation of the networks composed of various number of nodes. Different colours denote different advertisement intervals and operation modes. The plot on the right shows result for intermittent mode only as the PLR is significantly lower compared to continuous mode.

**Figure 7:**
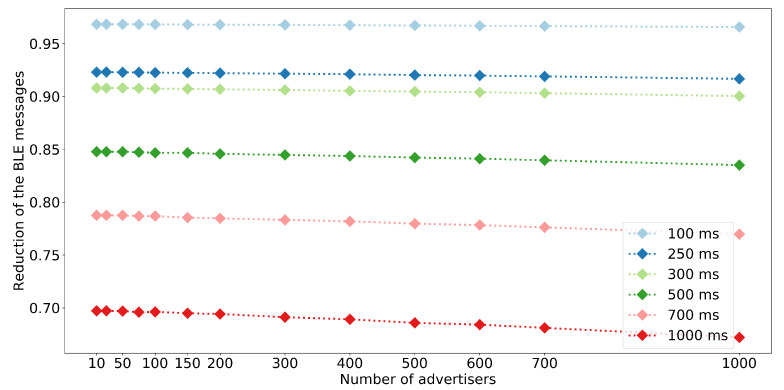
Median of the reduction of the number of BLE messages exchanged in continuous and intermittent mode for various advertisement intervals and network sizes. The reduction equals 1 minus ratio of ADV, SCAN_REQ and SCAN_RESP messages in intermittent mode versus the ADV messages in continuous mode.

Simulation results confirm real-life experiments. Figure 6 compares PLR for different advertisement intervals, number of nodes in the networks, and operation mode. The results for the network of 150 nodes are slightly different compared to experimental results (cf. Table 5). This is likely due to the fact that the simulation considers protocol related delays and collisions between the radio messages, and does not take into account all underperfomance of real-life devices. The results present that the intermittent operation significantly improves the PLR which is below 0.2 even for the network of 1000 nodes and advertisement interval of 100 ms. Moreover, in the intermittent mode the total number of ADVs, SCAN_REQs, and SCAN_RESPs transmitted does not depend on the network size and the advertisement interval, but is a function of the number of data intervals. Consequently, the total number of BLE messages transmitted in intermittent mode is notably lower compared to the continuous mode (Figure 7). For short advertisement intervals, the reduction exceeds 0.9 because of the high number of advertisements transmitted when nodes operate in continuous mode. Increasing advertisement interval lowers the number of ADV messages transmitted in continuous mode leading to the reduction of approximately 0.65 for the network of 1000 nodes and advertisement interval equal 1000 ms. Note that the reduction in the number of transmitted messages implies energy savings as time when nodes transmit messages is shorter.

The results confirm that intermittent operation can maintain the same DRR while significantly lowering both the number of BLE messages transmitted and PLR values. Consequently, intermittent operation allows to improve coexistence and fair spectrum sharing with other networks, as BLE nodes and scanners exchange much less radio messages compared to passive scanning and continuous operation.

**Figure 8:**
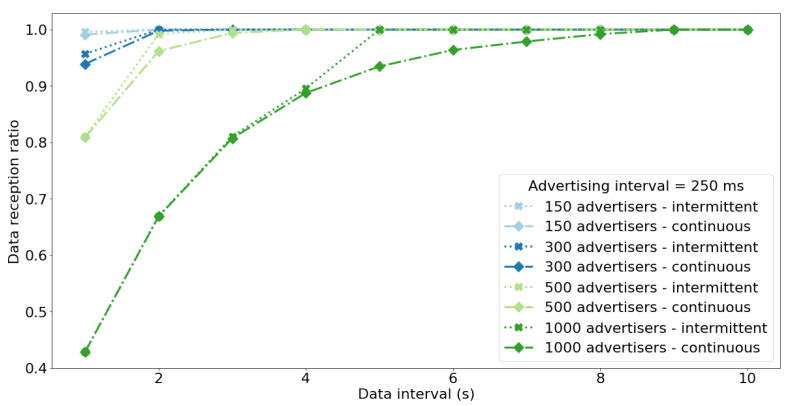
Median of the DRR${}_{\tau }$ for different data intervals τ, network sizes, and the advertisement interval equal 250 ms. For dense networks and short data intervals the DRR drops to the same values for both continuous and intermittent operation modes.

**Figure 9:**
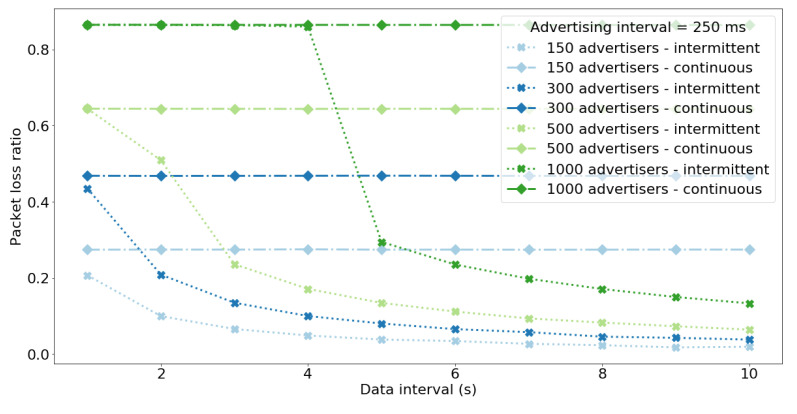
Median of the PLR for different data intervals τ, network sizes, and the advertisement interval equal 250 ms. The PLR for large networks and short data intervals is the same for nodes operating both in continuous and intermittent mode.

Figure 8 shows the value of data reception ratio for different values of data interval (DRR${}_{\tau }$), i.e., frequencies of data (sequence number) changes. The figure presents results for dense networks and frequent transmission of advertisements because for sparse networks or infrequent advertisements the DRR is always 1 for all data intervals. Considering the DRR${}_{\tau }$ for the network of 150 nodes and 250 ms advertisement interval it can be seen that the results are slightly higher compared to real-life experiments (Figure 5, Table 7) which is due to the simplified model of the nodes and scanner used in simulations. Despite simplifications, the results present that intermittent operation allows to significantly lower the data interval while maintaining high value of DRR. This means that the nodes can effectively transmit data more frequently and increase the effective data throughput when they operate in intermittent mode. For nodes operating in intermittent mode the DRR drops for dense networks, frequent transmission of advertisements, and short data intervals. This is because of the large PLR for those networks (Figure 9). In such scenarios the nodes restart ADV transmission frequently (due to small values of data interval) and experience lots of collisions (due to large number of nodes and small values of advertisement interval). As a result, the nodes effectively spent most of the time actively transmitting even it they operate in intermittent mode. Consequently, for dense networks with short data intervals and advertisement intervals, the benefits of using intermittent operation diminish and the network performs the same as for continous operation mode.

The results of experiments and simulations show, that the use of active scanning and intermittent transmission allows to reduce communication overhead, collisions in the communication channel, and keep the DRR high even for small values of the data interval. This allows to transmit greater amounts of data within the same time (increase effective throughput), or rise the number of BLE nodes in the system.

## 6. Conclusions

This article presents the benefits of using intermittent operation of BLE nodes in a typical IoT scenario, namely a dense network of large number of nodes, sensing and communicating periodically to the gateway. The real-life experiments with 150 nodes and thorough evaluation in simulation show that active scanning and intermittent operation of nodes can significantly reduce the number of BLE messages transmitted between the devices and lower the packet loss rate while all data is successfully transmitted. These improvements allow the battery-powered nodes to spend more time with radios disabled, preserve the energy and extend the lifetime. Less radio communications leaves more bandwidth to the other radios operating in a 2.4 GHz spectrum, thus becoming more fair in spectrum access. Moreover, because the nodes suspend transmission of ADV messages only after successful reception of SCAN_REQ, the proposed transmission scheme is immune to transient external interference including those caused by other radios consuming to much of the available bandwidth. The presented communication scheme follows the BLE specification and its implementation complexity is relatively small.

## Figures and Tables

**Table 1:** Settings of the BLE nodes and scanners, and corresponding communication modes for connection-less BLE communication using legacy advertisements.

PDU Type	Scanner Mode	Scheme
any	passive	single message
ADV_NONCONN_IND, ADV_DIRECT_IND	active	single message
ADV_IND, ADV_SCAN_IND	active	three message

**Table 2:** Symbols used in the article to describe operation of the BLE nodes and the scanners.

Symbol	Description
$T_{\mathrm{AI}}$	advertisement interval—the time between subsequent advertisement events. Each event is a transmission of 3 ADV messages and may contain exchange of SCAN_REQ and SCAN_RESP messages if active scanning is used
$t_{r}$	random time that is added to every advertisement interval
$T_{\mathrm{SI}}$	scan interval—the time between subsequent scanning operation initiated by the scanner. Every scan interval the scanner switches the radio channel used
$T_{\mathrm{SW}}$	scan window—the time scanner spends listening for ADV messages in every scan interval. The scan window is smaller or equal to scan interval. When both parameters are equal the scanner runs in continuous scanning mode.
$t_{d_{i}}$	the time when the first ADV messages containing the *i*th chunk of data is transmitted
$t_{a_{i}}$	the time when the scanner receives the ADV from a node transmitting the *i*th chunk of data
$t_{{\mathrm{req}}_{i}}$	the time when the node receives the SCAN_REQ message while sending ADV with *i*th chunk of data
$t_{{\mathrm{resp}}_{i}}$	the time when the scanner receives the SCAN_RESP message while the node is sending *i*th chunk of data

**Table 3:** Settings for the real-life experiment scenarios.

Scenario	Number of Nodes	Advertisement Interval (ms)	Data Interval (s)	Node Mode	Scanner Mode		
					S1	S2	S3
1	150	250	10	continuous	passive		
2	150	250	10	continuous	active	passive	
3	150	250	10	continuous	active		passive
4	150	250	10	intermittent	active	passive	

**Table 4:** Settings for the simulation scenarios.

Scenario	Number of Nodes	Advertisement Intervals (ms)	Data Intervals (s)
1	10, 50, 100, 150, 300, 500, 1000	100, 250, 500, 700, 1000	10
2	150, 300, 500, 1000	250	1, 2, 3, 4, 5, 6, 7, 8, 9, 10

**Table 5:** Statistics of ADV packets transmitted from the monitored node and received by scanners in different scenarios. PLR and DRR values are calculated for the monitored node.

Scenario	$N_{\mathrm{TxPacket}}$ per 10 s			Scanner	$N_{\mathrm{RxPacket}}$ per 10 s			PLR	DRR
	**Min**	**Median**	**Max**		**Min**	**Median**	**Max**	**Mean**	
				S1	2	12	26	0.686	1
1	39	40	40	S2	1	8	19	0.807	1
				S3	4	17	32	0.571	1
				S1	1	9	22	0.764	1
2	39	40	40	S2	1	6	15	0.85	1
				S3	5	14	30	0.639	1
				S1	2	12	26	0.705	1
3	39	40	40	S2	1	7	18	0.822	1
				S3	2	17	31	0.562	1
				S1	1	1	6	0.183	1
4	1	1	11	S2	0	1	7	0.592	0.565
				S3	0	1	9	0.123	0.942

**Table 6:** DRR and the number of packets received (in 10 s time windows) for all the 150 nodes in the experiments, different scenarios, and scanners. For scanners operating in the active mode the number of ADV and SCAN_RESP messages are given.

Scenario	Scanner	DRR			Total $N_{\mathrm{RxPacket}}$ in 10 s ADV/SCAN_RESP		
		**Min**	**Median**	**Max**	**Min**	**Median**	**Max**
1	S1	0.999	1	1	1524	1648	1799
	S2	0.993	1	1	1076	1285	1412
	S3	1	1	1	2131	2250	2361
2	S1	0.999	1	1	1299/595	1433/672	1562/775
	S2	0.994	1	1	983	1203	1308
	S3	0.999	1	1	2024	2162	2285
3	S1	0.999	1	1	920/432	1050/521	1148/604
	S2	0.997	1	1	651/300	831/408	913/482
	S3	1	1	1	1411	1560	1686
4	S1	1	1	1	155/122	172/139	236/174
	S2	0.51	0.625	0.763	85	119	161
	S3	0.685	0.937	0.953	162	204	276

**Table 7:** Estimates of DRR${}_{\tau }$ for scanner S1, various scenarios, and data intervals τ < 10 s. For scenarios 1–3, the estimates are calculated for all the 150 nodes in the experiments. For scenario 4, estimates are calculated for a single node which was monitored during the experiments.

Scenario	DRR${}_{\tau }$	Data Interval (τ) (s)								
		**1**	**2**	**3**	**4**	**5**	**6**	**7**	**8**	**9**
	min	0.591	0.826	0.917	0.955	0.97	0.985	0.985	0.992	0.992
1	median	0.757	0.932	0.985	0.992	1	1	1	1	1
	max	0.894	1	1	1	1	1	1	1	1
	min	0.553	0.78	0.886	0.932	0.962	0.977	0.977	0.977	0.985
2	median	0.697	0.902	0.962	0.985	0.992	1	1	1	1
	max	0.856	0.977	1	1	1	1	1	1	1
	min	0.527	0.747	0.835	0.868	0.89	0.901	0.912	0.912	0.912
3	median	0.714	0.901	0.965	0.978	0.989	0.989	0.989	0.989	0.989
	max	0.879	0.989	1	1	1	1	1	1	1
4		0.973	0.999	1	1	1	1	1	1	1
